# Factors Associated With Telemedicine Use Among Patients With Rheumatic and Musculoskeletal Disease: Secondary Analysis of Data From a German Nationwide Survey

**DOI:** 10.2196/40912

**Published:** 2023-01-27

**Authors:** Felix Muehlensiepen, Pascal Petit, Johannes Knitza, Martin Welcker, Nicolas Vuillerme

**Affiliations:** 1 Center for Health Services Research Faculty of Health Sciences Brandenburg Medical School Theodor Fontane Rüdersdorf bei Berlin Germany; 2 AGEIS Université Grenoble Alpes Grenoble France; 3 Department of Internal Medicine 3 Friedrich-Alexander-University Erlangen-Nürnberg and Universitätsklinikum Erlangen Erlangen Germany; 4 Medizinisches Versorgungszentrum für Rheumatologie Dr M Welcker GmbH Planegg Germany; 5 Institut Universitaire de France Paris France; 6 LabCom Telecom4Health, Orange Labs & Université Grenoble Alpes CNRS, Inria Grenoble INP-UGA Grenoble France

**Keywords:** telemedicine, rheumatology, primary care, secondary analysis, health services research

## Abstract

**Background:**

Previous studies have demonstrated telemedicine (TM) to be an effective tool to complement rheumatology care and address workforce shortage. With the outbreak of the COVID-19 pandemic, TM experienced a massive upswing. A previous study revealed that physicians’ willingness to use TM and actual use of TM are closely connected to their knowledge of TM. However, it remains unclear which factors are associated with patients’ motivation to use TM.

**Objective:**

This study aims to identify the factors that determine patients’ willingness to try TM (TM try) and their wish that their rheumatologists offer TM services (TM wish).

**Methods:**

We conducted a secondary analysis of data from a German nationwide cross-sectional survey among patients with rheumatic and musculoskeletal disease (RMD). Bayesian univariate and multivariate logistic regression analyses were applied to the data to determine which factors were associated with TM try and TM wish. The predictor variables (covariates) studied individually included sociodemographic factors (eg, age and sex) and health characteristics (eg, disease type and health status). All the variables positively or negatively associated with TM try or TM wish in the univariate analyses were then considered for the Bayesian model averaging analysis after a selection based on the variance inflation factor (≤2.5). All the analyses were stratified by sex.

**Results:**

Of the total 102 variables, 59 (57.8%) and 45 (44.1%) variables were found to be positively or negatively associated (region of practical equivalence ≤5%) with TM try and TM wish, respectively. A total of 16 and 8 determinant factors were identified for TM try and TM wish, respectively. Wishing that TM services were offered by rheumatologists, having internet access at home, residing 5 to 10 km away from the general practitioner’s office, owning an electronic device, and being aged 40 to 60 years were among the factors positively associated with TM try and TM wish. By contrast, not yet being diagnosed with an RMD, having no prior knowledge of TM, having a bad health status, living in a rural area, not documenting one’s health status, not owning an electronic device, and being aged 60 to 80 years were negatively associated with TM try and TM wish.

**Conclusions:**

Our results suggest that health status, knowledge, age, and access to technical equipment and infrastructure influence the motivation of patients with RMD to use telehealth services. In particular, older patients with RMD living in rural areas, who could likely benefit from using TM, are currently not motivated to use TM and seem to need additional TM support.

## Introduction

Telemedicine (TM) offers the opportunity to overcome spatial distances in health care delivery [1]. Therefore, given the increasing burden of musculoskeletal disorders worldwide [2] and the growing workforce shortage, especially in rural areas [3,4], TM does represent a promising opportunity to support rheumatology care [5,6]. However, the effective implementation of TM in standard care is only possible if end users are willing and able to use TM [7,8].

With the outbreak of the COVID-19 pandemic, face-to-face consultations by physicians decreased significantly [9,10]. The ability to provide noncontact medical care is now more important. Advantageously, TM can provide medical care with no risk of infection [11,12]. Therefore, TM has experienced a tremendous increase in use worldwide [13] and regionally [9,14]. Although the pandemic situation with social distancing and multiple lockdowns provided an ideal environment for TM implementation, this momentum soon stagnated again [10,15]. Especially in rheumatology, the use and acceptance of TM by health professionals fell short of expectations [10]. A recent secondary analysis of data from a physician survey found the knowledge of TM as a key factor in determining the willingness to use and actual use of TM among professionals [16]. However, this is only 1 side of the coin, and to successfully implement TM in standard rheumatology care, patients with rheumatic and musculoskeletal diseases (RMDs) must also be willing to try TM. The factors influencing this still need to be investigated and could have implications for the development of TM strategies aiming to improve health outcomes and access to care and make health care delivery systems more efficient and cost-effective.

To gain a better understanding of these factors, we performed a secondary analysis using data from a German nationwide cross-sectional survey conducted earlier [8]. Our objective was to identify the factors associated with patients’ will to try TM (TM try) and their wish that German rheumatologists offer TM services among patients with RMD (TM wish).

## Methods

### Overview

This work reports findings from a secondary analysis of data collected as part of a cross-sectional, self-completed, and paper-based survey of German patients with RMD in collaboration with the patient organization German League Against Rheumatism (Deutsche Rheuma-Liga, Landesvertretung Brandenburg) and outpatient rheumatologists. The survey was embedded in a >2-year mixed methods study investigating the acceptance, opportunities, and obstacles to the implementation of TM [8]. This survey was conducted from September 1 to December 30, 2019. The exact methodology of the survey has been described previously [8].

### Data Selection or Population Considered

From the aforementioned German nationwide survey, a data set of 438 patients in total was analyzed (Table 1). The response rate for each of the 26 questions is listed in Table 1. Individuals who missed to answer questions on age (question [Q] 17); sex (Q18); TM try (Q11: “Would you like to try telemedicine?”); or TM wish (Q14: “Would you like your rheumatologist to offer you telemedicine services?”) were excluded from this study. Consequently, a total of 282 (282/438, 64.4%) and 270 (270/438, 61.6%) patients were analyzed for TM try and TM wish, respectively.

**Table 1:** Regression analysis—variables considered (N=438).

Variable			Modality		Response rate, n (%)
**Dependent variables**					
	Q^a^11: “Would you like to try telemedicine?”	2 categories: yes and no		314 (71.7)	
	Q14: “Would you like your rheumatologist to offer you telemedicine services?”	2 categories: yes and no		277 (63.2)	
**Independent variables**					
	Q1: “How far do you drive to your rheumatology doctor’s office?”	8 categories: up to 10 km, 10-20 km, 20-30 km, 30-40 km, 40-50 km, 50-60 km, >60 km, and not answered		428 (97.7)	
	Q2: “How far do you drive to your GP^b^’s office?”	8 categories: up to 5 km, 5-10 km, 10-15 km, 15-20 km, 20-25 km, 25-30 km, >30 km, and not answered		434 (99.1)	
	Q3: “Have you ever contacted your doctor’s office using an electronic means?”	3 categories: yes, no, and not answered		431 (98.4)	
	Q4: “Do you own an electronic device?”	3 categories: yes, no, and not answered		434 (99.1)	
	Q5: “Do you have internet access at home?”	3 categories: yes, no, and not answered		434 (99.1)	
	Q8: “Prior to this survey, have you ever heard the term ‘telemedicine’?”	4 categories: yes, no, do not know, and not answered		409 (93.4)	
	Q11: “Would you like to try telemedicine?” (Q14 is considered as the dependent variable)	4 categories: yes, no, do not know, and not answered		314 (71.7)	
	Q14: “Would you like your rheumatologist offer you telemedicine services?” (Q11 is considered as the dependent variable)	4 categories: yes, no, do not know, and not answered		277 (63.2)	
	Q16: “Do you document your health status?”	4 categories: yes, on paper; yes, digitally; no; and not answered		402 (91.8)	
	Q17: age (continuous variable)	5 categories: <20 yo^c^, 20-40 yo, 40-60 yo, 60-80 yo, and >80 yo		422 (96.3)	
	Q18: sex	2 categories: female and male		425 (97)	
	Q19a: “RMD (Rheumatoid arthritis)”	3 categories: yes, no, and not answered		418 (95.4)	
	Q19b: “RMD (Spondylparthritis)”	3 categories: yes, no, and not answered		418 (95.4)	
	Q19c: “RMD (Psoriatic arthritis)”	3 categories: yes, no, and not answered		418 (95.4)	
	Q19d: “RMD (Collagenosis & Vasculitidis)”	3 categories: yes, no, and not answered		418 (95.4)	
	Q19e: “RMD (Arthrosis)”	3 categories: yes, no, and not answered		418 (95.4)	
	Q19f: “RMD (Crystal arthropathies)”	3 categories: yes, no, and not answered		418 (95.4)	
	Q19g: “RMD (Osteoporosis)”	3 categories: yes, no, and not answered		418 (95.4)	
	Q19h: “RMD (Fibromyalgia)”	3 categories: yes, no, and not answered		418 (95.4)	
	Q19i: “RMD (other)”	3 categories: yes, no, and not answered		418 (95.4)	
	Q19j: “RMD (not yet diagnosed)”	3 categories: yes, no, and not answered		418 (95.4)	
	Q19k: “RMD (do not know)”	3 categories: yes, no, and not answered		418 (95.4)	
	Q19l: “RMD (does not apply)”	3 categories: yes, no, and not answered		418 (95.4)	
	Q20: “How do you rate your health status?”	6 categories: very good, good, okay, bad, very bad, and not answered		417 (95.2)	
	Q21: “Are you in rheumatology treatment?”	4 categories: yes; no, I am a new patient: do not know: and not answered		426 (97.3)	
	Q23: “My place of residence is...”	5 categories: city (>100,000 inh^d^), town (20.000-100,000 inh), provincial town (5000-20,000 inh), rural area (<5000 inh), and not answered		420 (95.9)	

^a^Q: question.

^b^GP: general practitioner.

^c^yo: years old.

^d^inh: inhabitants.

### Regression Analysis

Both Bayesian univariate and multivariate logistic regression analyses were applied to the data to determine which factors were associated with TM try (Q11) and TM wish (Q14), respectively. In total, 26 independent variables were considered in each univariate regression analysis (Table 1). For questions other than Q11, Q14, Q17, and Q18, missing values (no answer) were considered a new category in the univariate regression analysis [17]. For instance, Q4 (“Do you own an electronic device?”) previously had 2 categories but was considered with 3 (yes, no, and not answered) in the univariate regression analysis. For statistical analysis, all the categorical variables having >2 modalities (eg, “yes,” “no,” and “do not know”) were transformed into dummy or binary variables. For instance, Q4 was transformed into 3 dummy variables. Age was considered as both a continuous and categorical variable.

**Figure 1:**
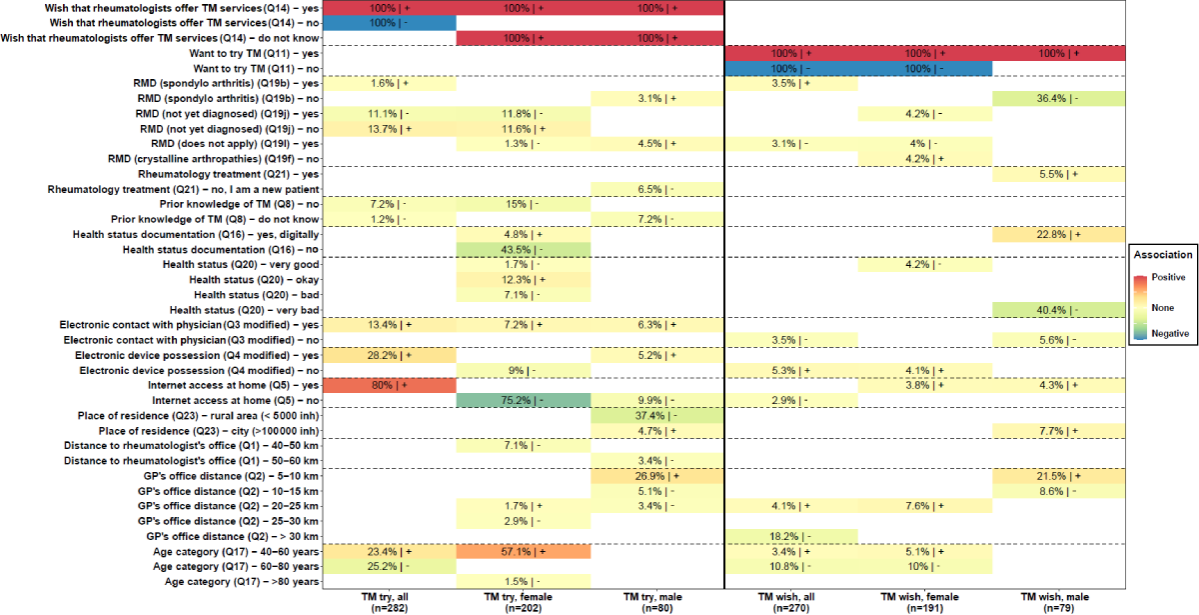
Determinants of patients’ willingness to try telemedicine (TM try) or patients’ wish that their rheumatologists offer telemedicine services (TM wish) identified through the Bayesian model averaging analysis. GP: general practitioner; inh: inhabitants; Q: question; RMD: rheumatic and musculoskeletal disease.

**Figure 2:**
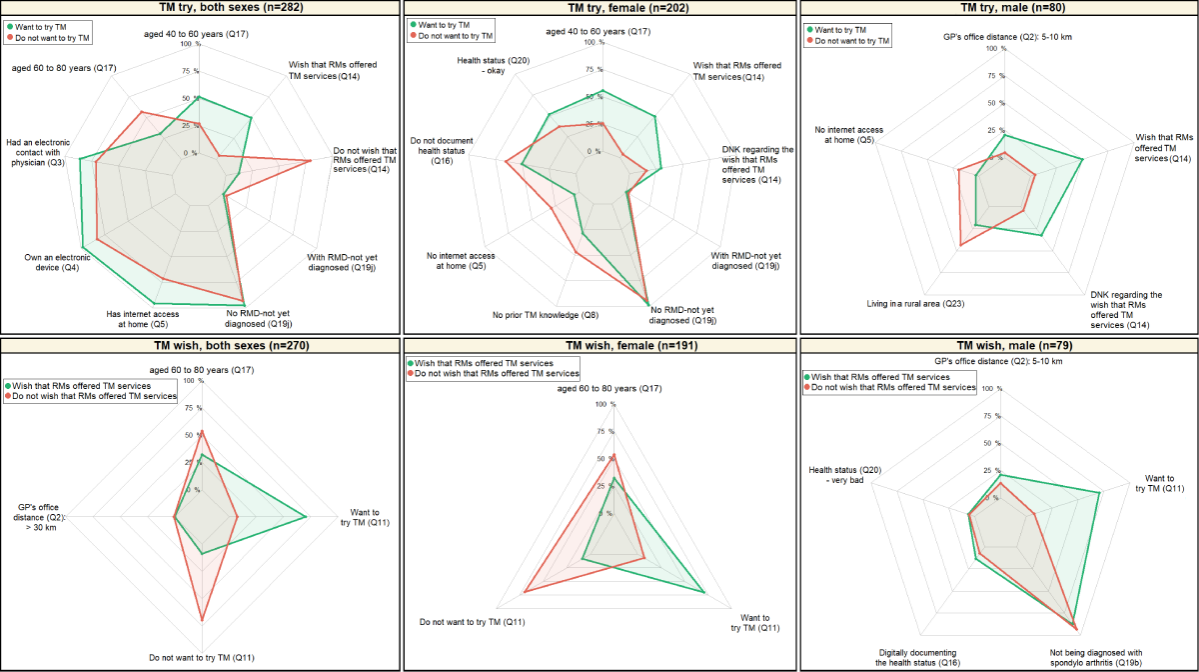
Profile of patients with rheumatic and musculoskeletal disease (RMD) who are motivated to try telemedicine (TM) versus that of patients with RMD who are not motivated to try TM. DNK: do not know; GP: general practitioner; RM: rheumatologist.

For each model, odds ratios (ORs) with 95% credible interval (CI) have been presented in Figures 1 and 2 and Multimedia Appendices 1-6. All the individual variables associated (positively or negatively) with TM try and TM wish in the Bayesian univariate analysis were analyzed in the later Bayesian multivariate analysis (model selection) after variable selection. This variable selection was based on the region of practical equivalence (ROPE) percentage (ROPE%≤5) [18-20], and a subsequent selection was based first on the variance inflation factor (VIF) [21]. Collinear covariates, with a VIF >2.5, were excluded from the multivariate models [22]. Finally, the determinants of TM try and TM wish were identified using Bayesian model averaging (BMA) [23]. All the models were stratified by sex.

All the statistical analyses were performed using R software (version 4.1.2, R Core Team) for Windows (version 10, Microsoft Corporation). Bayesian estimation was performed using the *rstanarm* package (version 2.21.1) [24,25]. Weakly informative priors (default priors in *rstarnarm*) were used. The default priors in *rstanarm* 2.21.1 are designed to be weakly informative. The Bayesian model adds priors (independent by default) to the coefficients of the generalized linear model. The Bayesian estimation was performed via the Markov chain Monte Carlo Bernoulli model, with 4 randomly initialized Markov chains, each for 2000 iterations (including a warm-up period of 1000 iterations that is discarded). BMA was performed using the *BMA package* (version 3.18.15) [26]. Regarding priors for BMA, we assumed that all candidate models were equally likely a priori (same prior weight).

### Ethical Considerations

Primary data collection was conducted in compliance with the current data protection regulations of the General Data Protection Regulation [27] and the Helsinki declaration. All the study participants were informed about the research project. Sending the questionnaire back to the study center was considered consent. Data were anonymized before analysis. The ethics committee of the Theodor Fontane Medical School in Brandenburg stated that no written consent was necessary owing to the noninterventional study design, which also applies to the secondary analysis.

## Results

### Population Characteristics

The response rate for the 26 questions ranged from 63.2% (277/438 for Q14) to 99.1% **(**434/438 for Q2, Q4, and Q5; Table 1). Among the 438 patients from the nationwide survey, 35.6% (n=156) were excluded from the TM try analysis because of missing data regarding age (n=16, 3.7%) or sex (n=13, 3%) or missing answer to Q11 (n=124, 28.3%). As for TM wish, a total of 168 (38.4%) patients were excluded from the analysis. Regardless of the analysis considered (TM try or TM wish), females represented 70.5% (n=309) of all the patients. In both TM try and TM wish analyses, rheumatoid arthritis was the most commonly represented RMD (143/282, 50.7% and 143/270, 53% of the patients, respectively), followed by arthrosis (65/270, 24.1% and 74/282, 26.2%, respectively), other RMDs (47/282, 16.7% and 43/270, 16%, respectively), osteoporosis (41/282, 14% and 39/270, 14%, respectively), psoriatic arthritis (34/282, 12.1% to 40/270, 14.8%, respectively), and spondyloarthritis (18/270, 7% and 21/282, 7%, respectively).

### Bayesian Univariate Logistic Regression Analysis

Of the total 102 variables, 59 (57.8%) and 45 (44.1%) variables (answers to the 26 questions) were found to be positively or negatively associated (ROPE%≤5%) with TM try and TM wish, respectively (Multimedia Appendices 1-6). After removing collinear variables (VIF >2.5), a total of 32 (31.4%) and 22 (21.6%) variables were considered in the BMA analysis for TM try and TM wish, respectively.

### BMA Analysis

**Figure 3:**
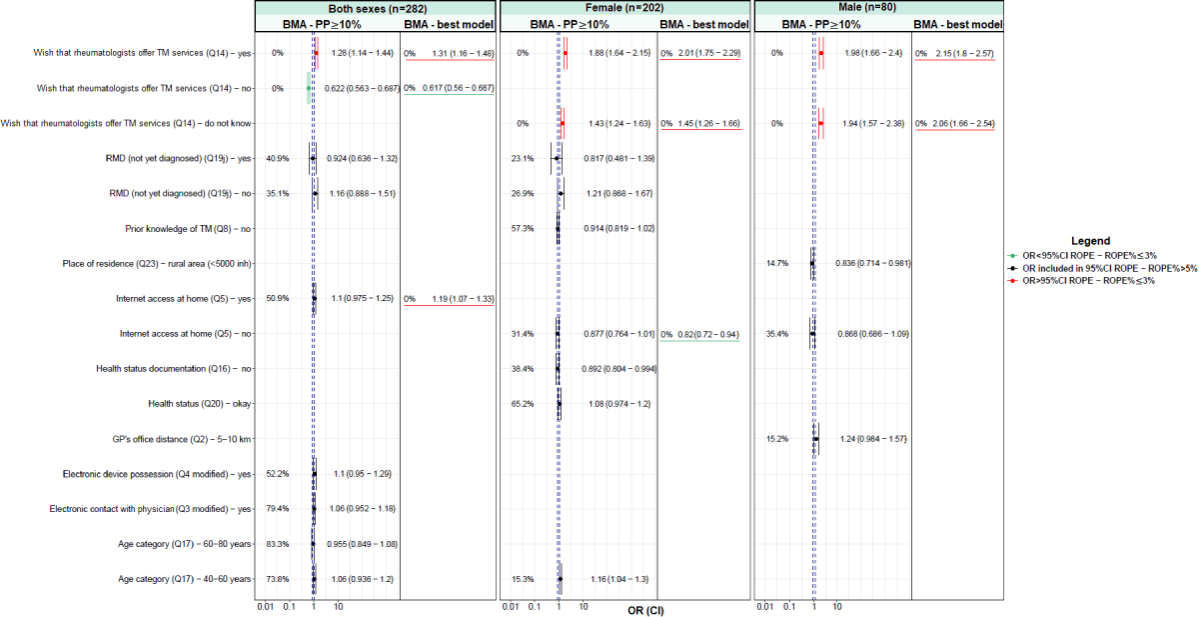
Bayesian multivariate logistic regression regarding patients’ willingness to try telemedicine (TM try)—results for variables with a posterior probability (PP) of ≥10% and for the best model identified with Bayesian model averaging (BMA). CI: credible interval; GP: general practitioner; inh: inhabitants; OR: odd ratio; Q: question; RMD: rheumatic and musculoskeletal disease; ROPE: region of practical equivalence.

A total of 6 BMA analyses were conducted, with 3 (both sexes, male and female) for TM try and 3 for TM wish. Figure 3 presents the factors identified through BMA for the 6 analyses. The value in each cell corresponds to the posterior probability that the considered variable is nonzero (in percentage). The darker the color, the higher the posterior probability percentage. Cells with colors from light yellow to red and the “+” sign refer to factors positively associated with TM try or TM wish. By contrast, cells with colors from light green to dark blue and the “-” sign refer to factors negatively associated with TM try or TM wish. Only variables with a posterior probability of ≥10% were considered determinant factors.

Regarding TM try, a total of 16 determinant factors were identified. Wishing that TM services were offered by a rheumatologist, having internet access at home, indicating a sufficient health status, residing 5 to 10 km away from the general practitioner’s (GP’s) office, being diagnosed with an RMD, owning an electronic device, having prior electronic contact with a physician, and being aged 40 to 60 years were positively associated with TM try. By contrast, not wishing that TM services were offered by a rheumatologist, not yet being diagnosed with an RMD, having no prior knowledge of TM, leaving in a rural area, not having internet access at home, not documenting one’s health status, not owning an electronic device, and being aged 60 to 80 years were negatively associated with TM try.

Regarding TM wish, a total of 8 determinant factors were identified. Wanting to try TM, living in a city, digitally documenting one’s health status, and residing 5 to 10 km away from the GP’s office were positively associated with TM wish. By contrast, not wanting to try TM, not being diagnosed with spondyloarthritis, stating to have a very bad health status, residing >30 km away from the GP’s office, and being aged 60 to 80 years were negatively associated with TM wish.

**Figure 4:**
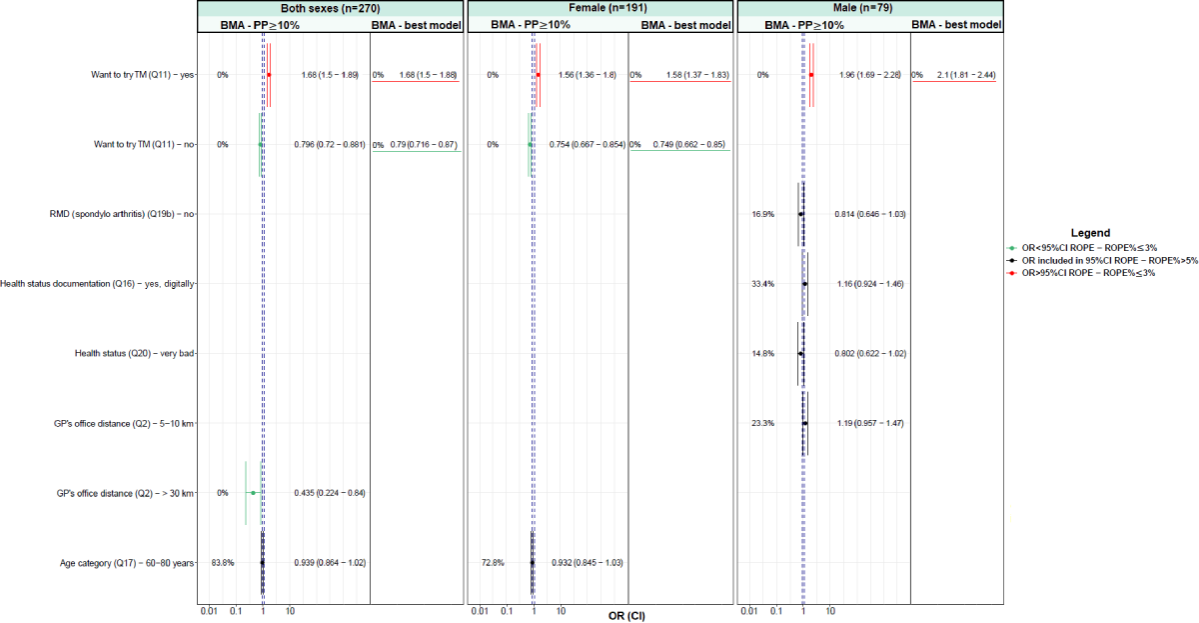
Bayesian multivariate logistic regression regarding patients’ wish that their rheumatologists offer telemedicine services (TM wish)—results for variables with a posterior probability (PP) of ≥10% and for the best model identified with Bayesian model averaging (BMA). CI: credible interval; GP: general practitioner; OR: odd ratio; Q: question; RMD: rheumatic and musculoskeletal disease; ROPE: region of practical equivalence.

Determinant factors identified with BMA (variables with a posterior probability of ≥10%) were used to establish the profile of patients with RMD who were motivated to use TM (TM try and TM wish) and that of patients with RMD who were not motivated to use TM. Figure 4 presents the profiles identified per sex. The variables displayed on the spider or radar chart correspond to the factors selected using BMA that had a posterior probability of ≥10%. The percentages refer to the percentage of patients with the answer specified for each question. For instance, 100% (the outer circular line, the farthest from the radar center) indicates that all patients answered the considered question with the specified answer (eg, being aged 40 to 60 years). By contrast, 0% (the inner circular line, the closest to the center) indicates that no patient chose the specified answer for the considered question (eg, being aged <20 years). The points indicate for each question the percentage of patients who chose the specified answer. Green points and lines refer to patients who wanted to try TM or wished that TM services were offered by their rheumatologists. Red points and lines correspond to patients who did not want to try TM and did not wish that TM services were offered by their rheumatologist. For each question, there were 3 possible situations. When the green and red points overlap (were similar), it means that there was no difference between patients whether they were motivated or not to use TM, and the proportion of similar answers were high. When the green point is higher (higher percentage) than the red point, it indicates that the patients motivated to use TM chose the specified answer more often than those not motivated to use TM, which means that this factor (answer to the question) had a positive impact on TM try or TM wish. Finally, when the green point is lower (lower percentage) than the red point, it indicates that the patients motivated to use TM chose the specified answer less often than those not motivated to use TM, which means that this factor (answer to the question) had a negative impact on TM try or TM wish.

Figures 1 and 2 present the results of the Bayesian multivariate logistic regression analysis considering the determinant factors (variables with a posterior probability of ≥10%) as well as the best model identified using BMA for TM try and TM wish, respectively.

Regarding TM try (Figure 1), the patients who wished that TM services were offered by their rheumatologists, those who did not know whether they wished that TM services were offered by their rheumatologist, and those who had internet access at home were associated with the willingness to try TM. By contrast, the patients who did not wish that TM services were offered by their rheumatologists were associated with less willingness to try TM.

Regarding TM wish (Figure 2), the patients who did not want to try TM and lived >30 km away from their GP’s office were associated with less desire for their rheumatologists to offer TM services. By contrast, the patients who wanted to try TM were associated with a greater desire for their rheumatologists to offer TM services.

In Figure 1, the percentage indicates the ROPE percentage, that is, the probability that the considered credible factor values are not negligible. The dashed lines indicate the ROPE 95% CI.

If the OR 95% CI is included (ROPE%>97.5%) in the ROPE 95% CI (dashed blue vertical lines), it means that there was no relationship between the considered factor and TM try. If the ROPE% is ≤3%, it means that the factor was significant for explaining TM try; otherwise, it was not (in black). When the ROPE% is ≤3% and the OR is inferior to the ROPE 95%, the considered factor had a significant negative effect on TM try (in green). By contrast, when the ROPE% is ≤3% and the OR is superior to the ROPE 95%, the considered factor had a significant positive effect on TM try (in red).

In Figure 2, the percentage indicates the ROPE percentage, that is, the probability that the considered credible factor values are not negligible. The dashed lines indicate the ROPE 95% CI.

If the OR 95% CI is included (ROPE%>97.5%) in the ROPE 95% CI (dashed blue vertical lines), it means that there was no relationship between the considered factor and TM wish. If the ROPE% is ≤3%, it means that the factor was significant for explaining TM wish, otherwise, it was not (in black). When the ROPE% is ≤3% and the OR is inferior to the ROPE 95%, the considered factor had a significant negative effect on TM wish (in green). By contrast, when the ROPE% is ≤3% and the OR is superior to the ROPE 95%, the considered factor had a significant positive effect on TM wish (in red).

## Discussion

We performed a secondary analysis using data from a German nationwide cross-sectional survey among patients with RMD [8]. Our objective was to identify the factors associated with TM try and TM wish to enable more effective TM strategies.

### Principal Findings

Our results revealed that the factors determining the motivation of patients with RMD toward using TM were multidimensional. The patients who wanted to try TM more frequently owned an electronic device, more often had internet access at home, and were aged between 40 and 60 years. The patients who did not want to try TM more often lived in rural areas, had less access to the internet at home, had no prior knowledge of TM, and did not document their health status. These results suggest that TM could cause a digital divide and is currently not supporting those who will benefit the most from it, such as patients who have to travel long distances and those who are not in a good health status. The patients who wished that TM services were offered by their rheumatologists were more often willing to try TM. By contrast, the patients who did not wish that TM services were offered by their rheumatologists were more often aged 60 to 80 years, living >30 km away from their GP’s office, in a bad health status, not being diagnosed with spondyloarthritis, and not willing to try TM.

### Comparison With Prior Work

To the best of our knowledge, this is the first study to analyze the specific factors influencing the motivation among German patients with RM to implement TM. The presented findings might inform public and private stakeholders to guide TM implementation strategies.

Our results indicated that especially in remote areas, where TM is considered to have the largest impact on health care delivery [28-30], patients with RMD are not motivated to use TM. Clearly, this is also linked to the regional technical infrastructure, specifically internet access, which is still inadequate and a major challenge in several remote regions in Germany [8]. In fact, our results underline that not only the availability of infrastructure but also the individual possession of technical equipment determines the willingness to implement TM. Those who do not possess technical devices will not use TM. These findings are in line with previous studies from other medical domains pointing to the digital divide and the danger of socioeconomic inequalities in the use of eHealth [31-33]. Furthermore, our data confirm a demographic divide in the use of eHealth and mobile health [32,34]: patients aged between 40 and 60 years were more willing to try TM, whereas those aged 60 to 80 years did not want their rheumatologists to offer them TM. Furthermore, similar to the corresponding finding among physicians [16], the knowledge of TM was an important determinant of TM try. In line with our results, Tennant et al [35] showed that being younger, using more electronic devices, and possessing a higher level of education positively influences eHealth literacy. Similarly, Knitza et al recently reported that being younger positively correlates with higher eHealth literacy [36] and higher usability ratings [37] among patients with RMD.

### Implications

Our results demonstrate that many patients with RMD will not have access to TM without further support. This is particularly problematic, as TM is expected to reduce the rheumatology workforce shortage and is, thus, increasingly implemented in rheumatology care delivery [38]. Provided that the digital transition [39] in rheumatology care continues, specific patient groups could be excluded from quality health care: older patients, those living in rural areas, those without adequate internet access, and those not possessing electronic devices because of lack of economic endowment. These patient groups require support for the use of digital rheumatology care. We strongly support the provision of high-quality and low-threshold information and support services for patients to transfer the knowledge of TM and digital health. Health insurance companies, patient organizations, and adult education centers could play a central role in this. In addition, Dahlhausen et al [40] recently pointed out that physicians are the most important in promoting TM (digital therapeutics) use among patients. However, in the secondary analysis of factors associated with TM use among general practitioners and rheumatologists, we found that characteristics similar to those in patients with RMD, specifically the lack of prior TM knowledge, age (51 to 60 years), and being located in rural areas, hamper physicians from using TM [16]. Programs to promote digital health competencies in rural areas could thus benefit from the involvement of both professional and patient organizations, thereby allowing interaction on optimal use, regional needs, and user preferences toward TM. However, financial incentives for physicians to promote TM are lacking: more than half of the participants of a nationwide survey among physicians reported poor reimbursement as a key barrier to the implementation of TM [7]. Concurrently, only 4% (25/675) of the patients with RMD surveyed in the initial study [8] reported that they were willing to pay for TM out of their pocket. This underlines the need for innovative reimbursement models that adequately compensate the TM and digital health services in Germany.

However, even as TM becomes fully entrenched into standard health care delivery, patients who are not willing or unable to use TM must continue to have the option of receiving traditional, nondigital health–supported care. Therefore, we support the approach by Kulcsar et al [41] of using a triage mechanism to ensure that patients are appropriately paired with the proper type of rheumatology care in the future.

### Limitations

The primary data on which this analysis was based were collected until December 30, 2019, that is, shortly before the SARS-CoV-2 outbreak in Germany (January 27, 2020). Owing to the need to reduce physical contact and thus minimize the risk of infection, the use of TM initially received a major uptake in global health care delivery [13]. Hence, more patients with RMD and likely other subgroups would have tried TM by now [42]. A replication of the initial survey is essential to examine whether and how the identified factors have changed. In addition, the limitations of the primary data still apply [8]. These are primarily the high potential for self-selection and nonresponse bias. In addition, we cannot exclude a selection bias because individuals with missing data regarding age (16/438, 3.7%) or sex (13/438, 3%) were excluded from this analysis. However, the missing data for each variable of interest represented less than 10% of the total data. According to Langkamp et al [43], in this situation, the introduced bias is slight.

Sex skewness, with 70.5% (309/438) of the population studied being females, could be a limitation. However, to address this potential limitation, the analyses were stratified based on sex to identify potential sex differences related to TM try and TM wish. In addition, females are more often affected by RMDs than males [44]; therefore, the data roughly reflect the sex ratio in the target population.

In statistical analyses, we used a Bayesian approach to conduct a secondary analysis of the aforementioned survey. A practical limitation of the Bayesian approach is that it requires the specification of prior distributions on both the parameters of each model and distribution of the models themselves. As we had no a priori assumption, we used weakly informative priors. Choosing another prior distribution may have had a substantial influence on the outcome [45,46]. Regarding variable selection, a widespread approach consisting of including significant variables from the univariate analysis in a multivariate analysis was carried out [47,48]. To be more accurate, all the individual variables associated (positively or negatively) with TM try or TM wish in the Bayesian univariate analysis were selected based on the ROPE percentage (ROPE%≤5%). A ROPE-only decision rule was used, as suggested in other works [18-20]. Choosing a different ROPE percentage threshold may have yielded different results. Then, we performed a conservative selection based on VIF (VIF ≤2.5) to deal with potential variable multicollinearity. Finally, we used the remaining variables with BMA for model selection and the identification of determinants. BMA was chosen in particular because it reduces overconfidence and is relatively robust against model misspecification [43,49-51]. Markov chain Monte Carlo was used to deal with the intractable computational challenge of BMA that comes from the candidate model enumeration [52].

### Conclusions

Specific subgroups of patients with RMD will not have access to TM or motivation for TM use without further support. These are older patients, those living in rural areas, those without adequate internet access, those in a bad health status, and those not possessing electronic devices owing to a lack of economic endowment. We strongly support the provision of high-quality and low-threshold information and support services for patients to foster TM use.

## Appendix

Multimedia Appendix 1 Bayesian univariate logistic regression—relationship between patients’ willingness to try telemedicine (TM try) and patient characteristics—part 1. GP: general practitioner; Q: question; ROPE: region of practical equivalence.

Multimedia Appendix 2 Bayesian univariate logistic regression—relationship between patients’ willingness to try telemedicine (TM try) and patient characteristics—part 2. Q: question; ROPE: region of practical equivalence.

Multimedia Appendix 3 Bayesian univariate logistic regression—relationship between patients’ willingness to try telemedicine (TM try) and patient characteristics—part 3. Q: question; RMD: rheumatic and musculoskeletal disease; ROPE: region of practical equivalence.

Multimedia Appendix 4 Bayesian univariate logistic regression—relationship between patients’ wish that their rheumatologists offer telemedicine services (TM wish) and patient characteristics—part 1. GP: general practitioner.

Multimedia Appendix 5 Bayesian univariate logistic regression—relationship between patients’ wish that their rheumatologists offer telemedicine services (TM wish) and patient characteristics—part 2. Q: question; RMD: rheumatic and musculoskeletal disease; ROPE: region of practical equivalence.

Multimedia Appendix 6 Bayesian univariate logistic regression—relationship between patients’ wish that their rheumatologists offer telemedicine services (TM wish) and patient characteristics—part 3. TM: Telemedicine; Q: question; ROPE: region of practical equivalence.

## Abbreviations

BMA: Bayesian model averaging
CI: credible interval
GP: general practitioner
OR: odds ratio
Q: question
RMD: rheumatic and musculoskeletal disease
ROPE: region of practical equivalence
TM: telemedicine
TM try: patients’ willingness to try telemedicine
TM wish: patients’ wish that their rheumatologists offer telemedicine services
VIF: variance inflation factor
