# Supplementary figures and images for: Factors Associated With Telemedicine Use Among Patients With Rheumatic and Musculoskeletal Disease: Secondary Analysis of Data From a German Nationwide Survey
Source: J Med Internet Res. 2023 Jan 27;25:e40912. doi: 10.2196/40912 (PMC9919449; doi:10.2196/40912)

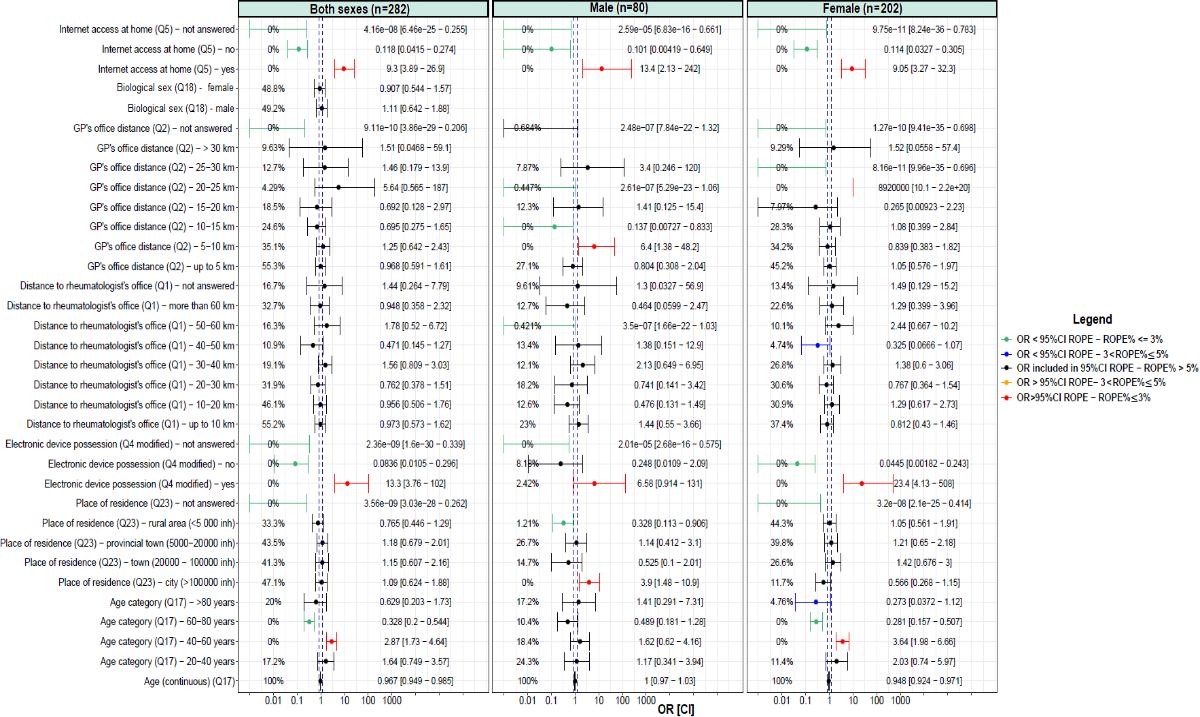

Supplement: Multimedia Appendix 1 [file jmir_v25i1e40912_app1.png]

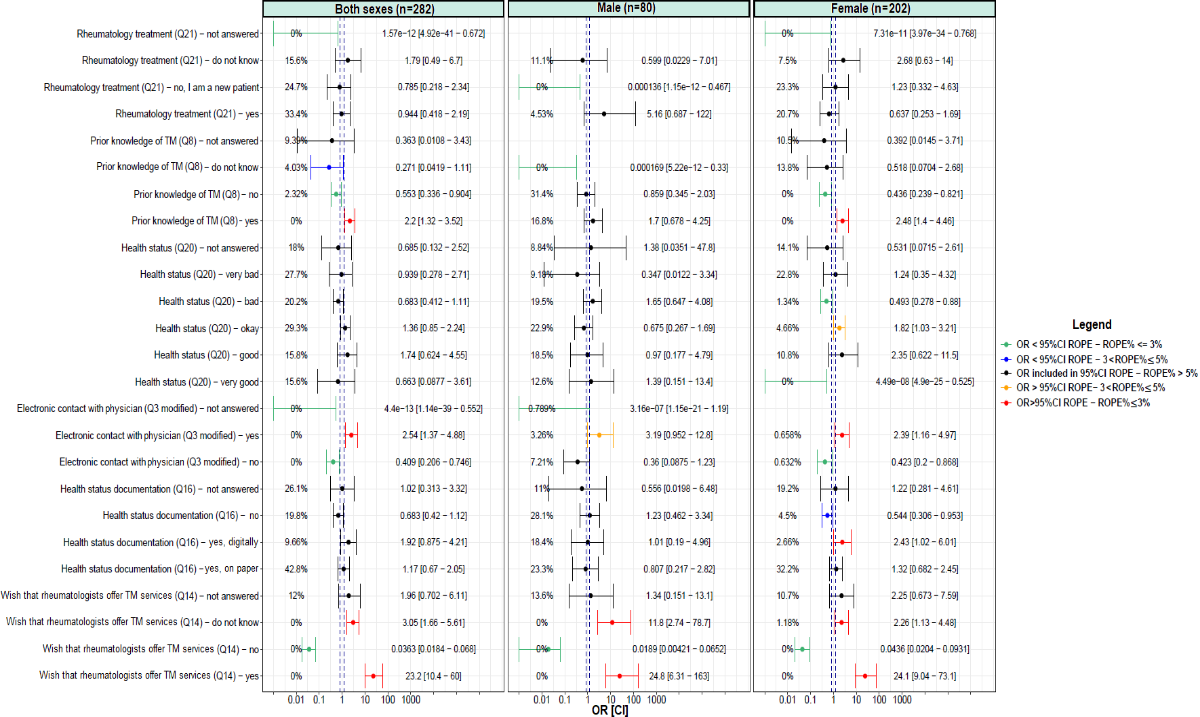

Supplement: Multimedia Appendix 2 [file jmir_v25i1e40912_app2.png]

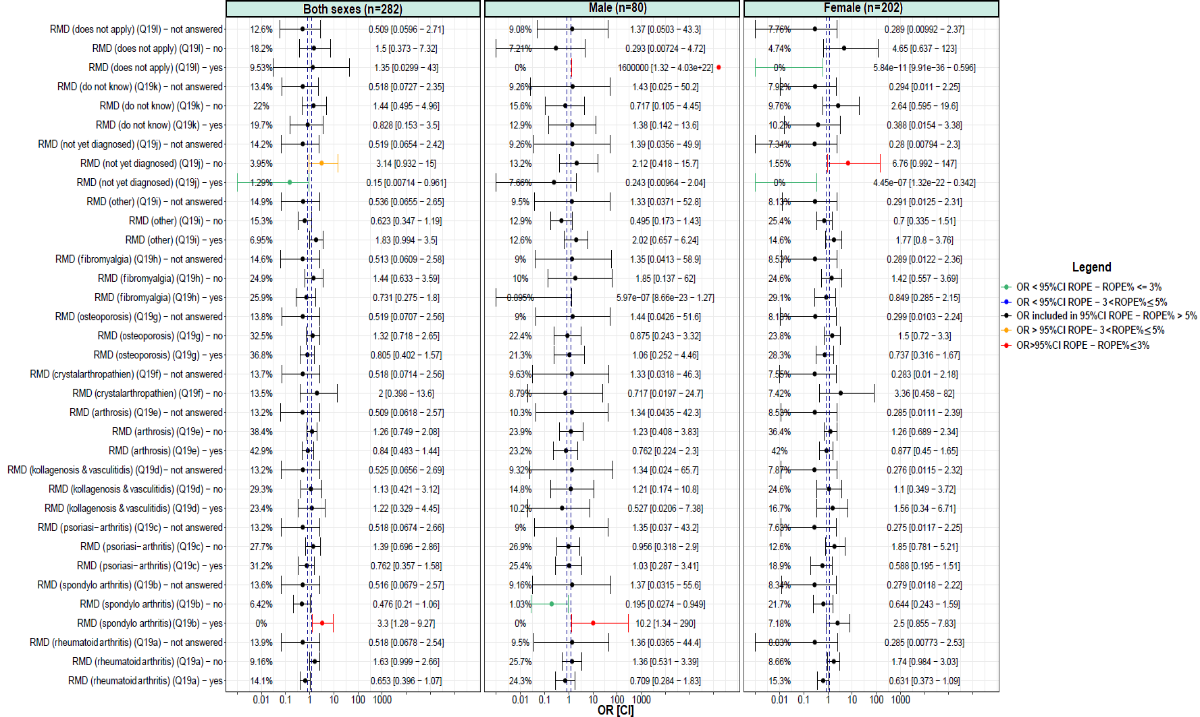

Supplement: Multimedia Appendix 3 [file jmir_v25i1e40912_app3.png]

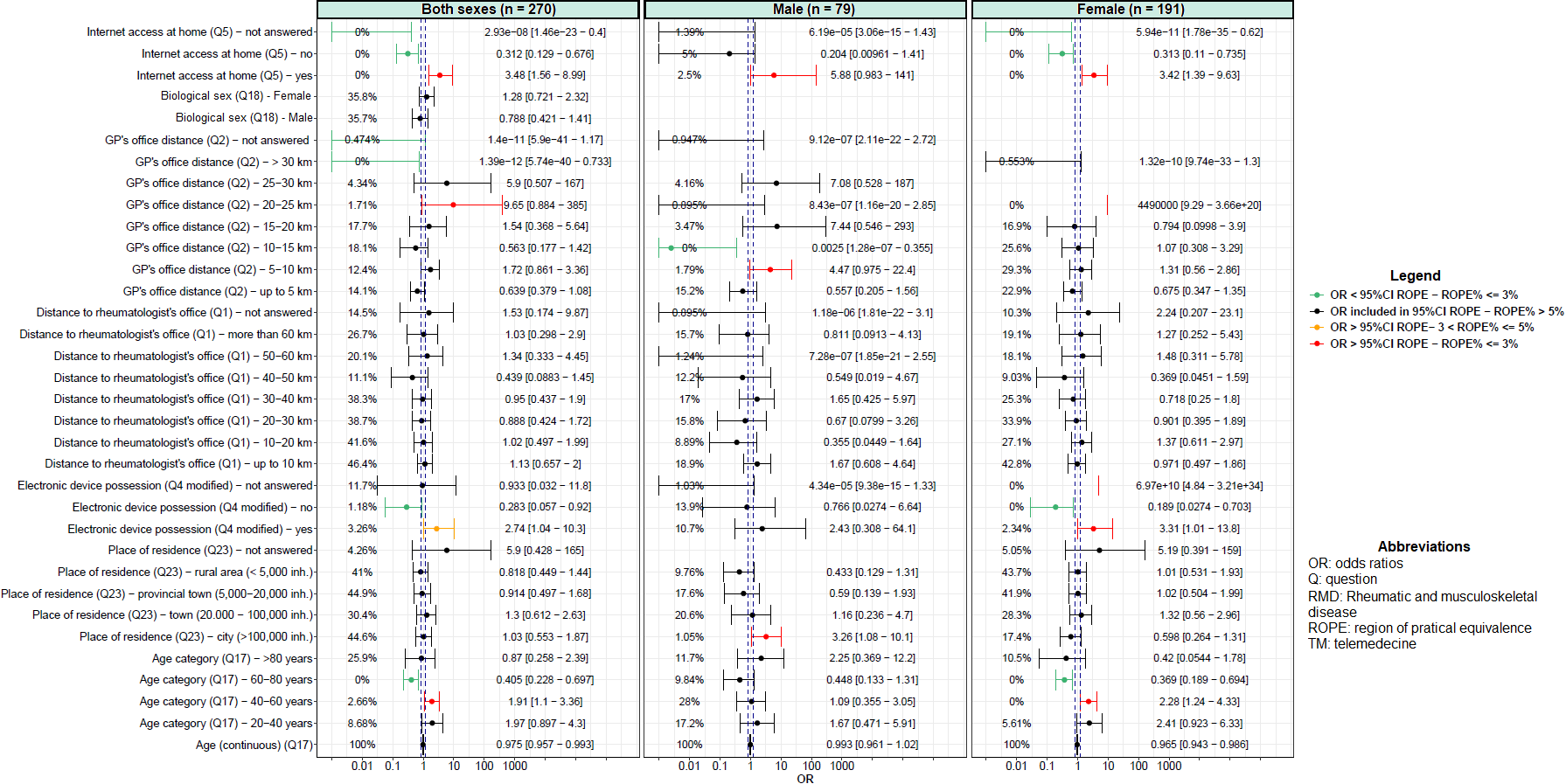

Supplement: Multimedia Appendix 4 [file jmir_v25i1e40912_app4.png]

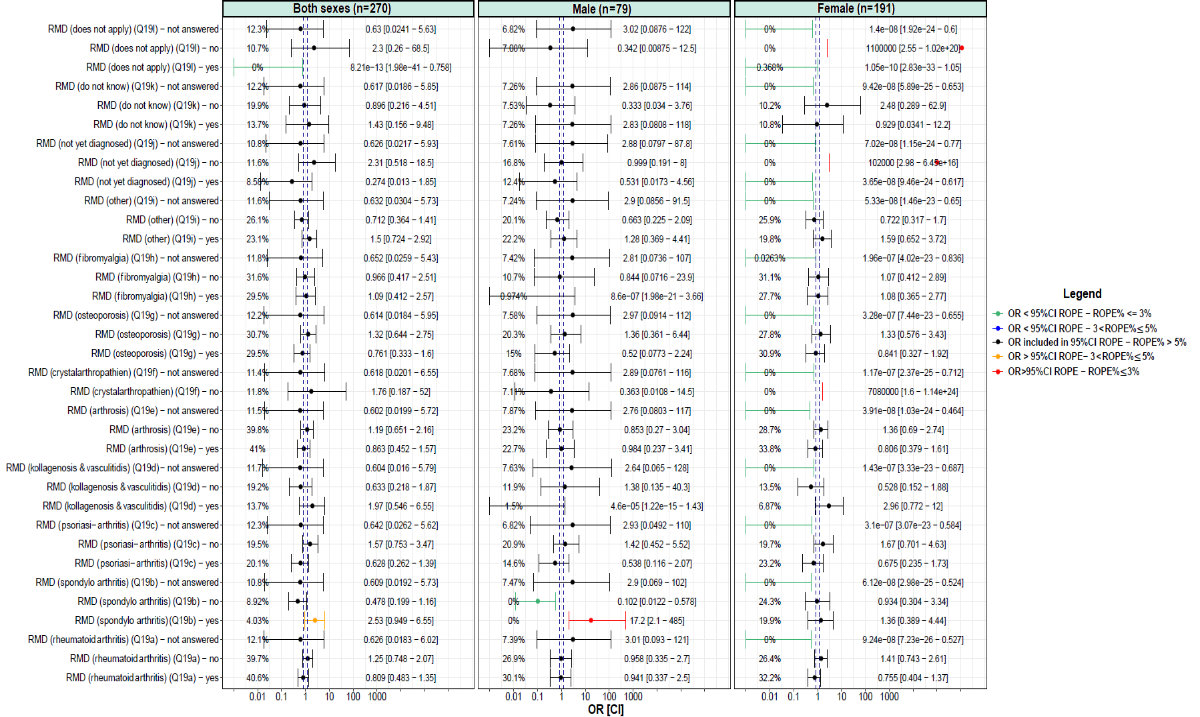

Supplement: Multimedia Appendix 5 [file jmir_v25i1e40912_app5.png]

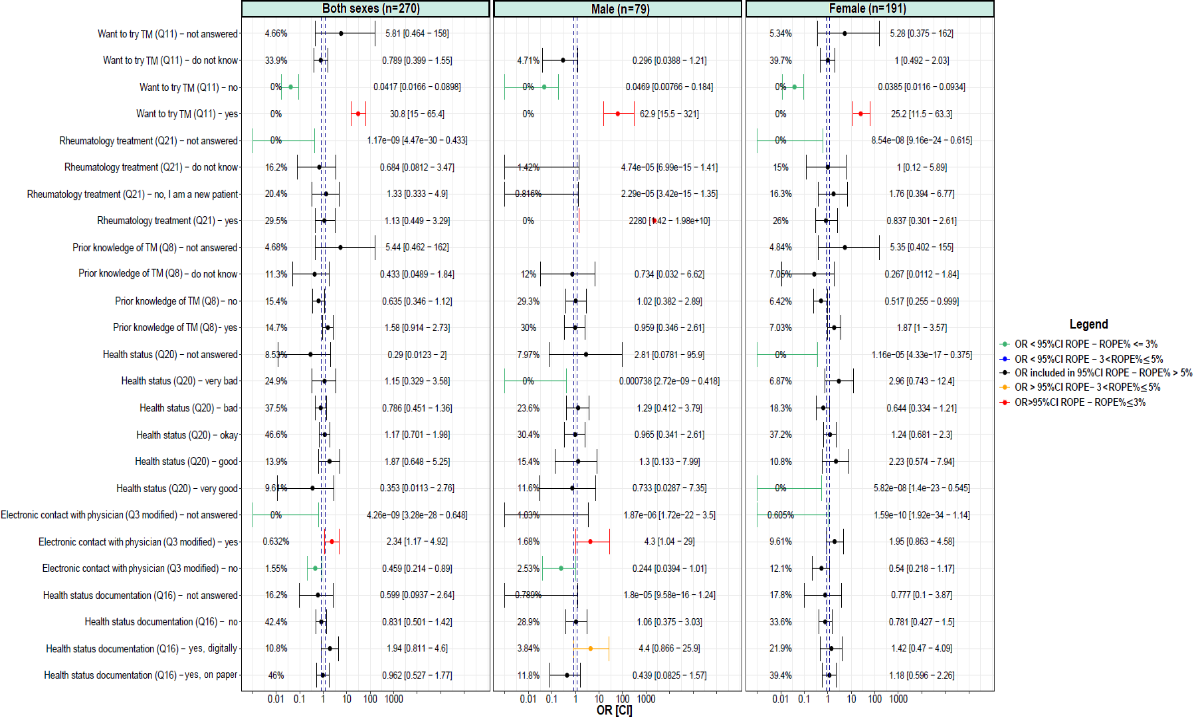

Supplement: Multimedia Appendix 6 [file jmir_v25i1e40912_app6.png]
